# Kleine Welt: Wenn Deutschland nur mit Demokratien handelt

**DOI:** 10.1007/s10273-022-3225-1

**Published:** 2022-07-23

**Authors:** Lukas Menkhoff

**Affiliations:** Abtl. Weltwirtschaft, Mohrenstr. 58, 10117 Berlin, Deutschland

**Keywords:** F13, F14

## Abstract

Die vergangenen Jahre haben die Kehrseite des deutschen Exportmodells verdeutlicht, die Abhängigkeit Deutschlands von seinen Handelspartnern. Besonders schmerzhaft ist dies gegenwärtig gegenüber Russland und potenziell gegenüber China. Insofern scheint es naheliegend, den deutschen Außenhandel auf demokratische Länder zu konzentrieren. Doch gleichgültig welchen Maßstab man wählt, die demokratische Welt ist klein und ihr Gewicht nimmt auch nicht zu. Zudem sind die weniger demokratischen Länder tendenziell ärmer, sodass der Handel eine spezifische Struktur aufweist. Es dominiert der Import von Rohstoffen, die in Deutschland und Europa kaum verfügbar sind. Entsprechend hoch wären die ökonomischen Kosten solch einer Selbstbeschränkung.

## Abstract (en)

The last few years have shown the downside of the German export model, i. e. Germany’s dependency on its trading partners. This is particularly painful at present with regards to Russia and potentially China. Thus it seems reasonable to focus German trade on democratic countries. However, whatever benchmark one takes, the democratic world remains small and does not increase in importance. Moreover, the less democratic countries are tentatively poorer which implies that their trade structure follows a specific pattern; in particular these countries have a higher share of commodity exports which are hardly available in Germany or Europe. Consequently, the economic costs of such a self-imposed restriction would be high.

Der Außenhandel ist heute weltweit viel stärker mit politischen Ambitionen verknüpft als er dies vor zehn oder 20 Jahren war. Die Hochphase der Globalisierung in den vergangenen Jahrzehnten war von einer Öffnung neuer Märkte (vor allem in Asien und Osteuropa), sinkenden Transaktionskosten und vergleichsweise ungestörten Handelsbeziehungen gekennzeichnet. Dies hat sich speziell seit der großen Wirtschaftskrise 2008/2009 geändert; der internationale Austausch wird durch Sanktionen, steigende Zölle und Eingriffe in die Investitionsfreiheit gebremst (Görg und Kamin, 2021). Manchmal wird auch, positiv gewendet, Selbstversorgung angestrebt (Dieter, 2021). Außenhandel hat neben der ökonomischen eine politische Dimension bekommen.

## Handelsbeschränkungen auch durch Deutschland

In Deutschland denkt man bei politisch motivierten Handelsbeschränkungen zuerst an die Politik der letzten US-Regierung oder an China, aber nicht an Deutschland selbst. Entsprechend kritisch ist die Sicht auf die USA, zumal hier die aktuelle Regierung die Zollerhöhungen der Trump-Administration im Kern nicht zurückgenommen hat, obwohl sie rein ökonomisch den USA und den meisten anderen Ländern schaden (z. B. Boer et al., 2021). Noch kritischer wird China betrachtet, weil es selbst Märkte abschottet, aber von eigenen Rekordexporten profitiert.

Tatsächlich hat aber auch in Deutschland bzw. Europa der Handel eine stärker politische Dimension bekommen, auch schon vor den jüngsten schwerwiegenden Sanktionen gegen Russland. Offensichtlich ist dies bei den Sanktionen gegen den Iran, den Beschränkungen von Rüstungsexporten oder bei der immer umfangreicher werdenden Liste deutscher Industriezweige, bei denen deutsche Sicherheitsinteressen ausländische Investitionen beschränken, was sich in erster Linie auf China bezieht (Felbermayr und Görg, 2020). Allgemeiner als solche länderbezogenen Maßnahmen ist das „Lieferkettengesetz“, das deutsche Firmen verpflichtet, die Einhaltung sozialer und ökologischer Mindeststandards über ihre gesamte internationale Lieferkette hinweg sicherzustellen. Was also strebt die Politik an? Wohin würde es führen, wenn Deutschland nicht mehr mit der ganzen Welt Handel treibt, sondern nur noch mit Ländern, die ähnliche Werte teilen? Wobei dies hier als ähnlich „demokratisch“ verstanden wird. Welche Länder dies sind, hängt davon ab, wie strikt die Definition eines demokratischen Landes gefasst wird.

## Kleine Welt der guten Demokratien

Unterstellt man einmal, Deutschland würde sehr rigorose Anforderungen stellen und nur noch mit Ländern Außenhandel betreiben, deren Staatsform eine Demokratie nach unse-

* Der Verfasser dankt Lukas Boer und Christopher Kißling für wertvolle Unterstützung.

**Prof. Dr. Lukas Menkhoff** lehrt Volkswirtschaftslehre an der Humboldt-Universität zu Berlin und leitet die Abteilung Weltwirtschaft am DIW Berlin.

rem Verständnis ist, dann reduziert man die Partner qualitativ – ohne hier schon auf exakte Werte für das jeweilige Maß an Demokratie einzugehen – im Kern auf West- und Mitteleuropa, Nordamerika und wenige Länder im pazifischen Raum. Um dies zu quantifizieren, kann man den VA-Index der Weltbank heranziehen, der „Voice and Accountability“ im Verhältnis zwischen Bürger:innen und Regierungen misst. Der Index erfasst also das Ausmaß, in dem Bürger:innen eines Landes in der Lage sind, sich an der Wahl ihrer Regierung zu beteiligen sowie die Funktionsfähigkeit der dabei wichtigen Mediatoren; das sind Meinungsfreiheit, Versammlungsfreiheit und freie Medien. Dieser Demokratieindex setzt sich aus einer Reihe von Teilindices zusammen. Sicher kann man sowohl über die Berücksichtigung der Teilindices sowie das gewählte Aggregationsverfahren unterschiedlicher Meinung sein. Allerdings hängt das qualitative Ergebnis nicht von solchen Entscheidungen ab, wie später gezeigt wird. Der VA-Index hat den weiteren Vorteil, breit verfügbar zu sein, d. h. für 203 Länder im Vergleich zu 240 Ländern, die bei der Weltbank generell statistisch erfasst werden. Die Differenz wird nur von Kleinststaaten gebildet, in Europa z. B. von Gibraltar oder dem Vatikanstaat. So repräsentieren die 203 Länder 98 % der Weltbevölkerung bzw. 99 % des Welteinkommens und der deutschen Exporte.

In Abbildung 1 ist die Verteilung der Indexwerte für die 203 erfassten Länder 2020 abgebildet, die aufgrund der Konstruktion des VA-Index Werte von -2,5 bis 2,5 aufweisen können. Faktisch liegt der niedrigste Wert für Nordkorea bei -2,16 und der höchste für Norwegen bei 1,73. Deutschland liegt mit einem VA-Wert von 1,38 am oberen Rand der Verteilung. Es zeigt sich, dass nur ein kleiner Teil der Welt einen VA-Index aufweist, der dem deutschen ähnlich ist.

## Verschiedene Anforderungen an gute Demokratien

Wie stark würde sich nun eine Beschränkung des Außenhandels auf Demokratien auswirken? Das hängt auch davon ab, welcher Grad an guter Demokratie als akzeptabel empfunden wird, denn entsprechend verändert sich die Gruppe der demokratischen Länder. Im Folgenden wird deshalb die Festlegung des Mindestgrads an guter Demokratie nach dem VA-Index in vier Schritten definiert:

(i) Demokratien müssen so „gut“ wie die deutsche sein, gemessen am Wert des Jahres 2020 (VA-Wert 1,38). Dies beinhaltet – wie jede der folgenden „Mindestanforderungen“ – eine gewisse Willkür und (wegen des einmaligen Zeitpunkts 2020) auch Zufälligkeit. Diesen Wert erreichen 2020 nur wenige EU-Staaten, selbst die alten Demokratien Großbritannien (1,25) und Frankreich (1,07) haben einen VA-Wert darunter.

(ii) Demokratien müssen einen Wert mindestens so hoch wie der niedrigste Wert eines EU-Staates vor der Osterweiterung ab 2004 aufweisen, das ist Griechenland mit einem aktuellen VA-Wert von 0,97. Diese Demokratien werden als ähnlich gefestigt und funktional wie die deutsche eingeschätzt. Dazu zählen auch die tschechische Republik und Japan, um eine Idee über die Konsequenz dieses Standards zu bekommen.

Abbildung 1

**Ausprägung des Demokratieindex 2020**

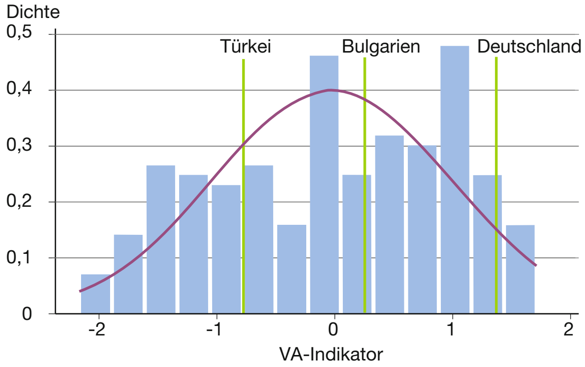

Der Grad der Demokratie im jeweiligen Land wird durch den VA-Index der Weltbank (2022) erfasst. Die Türkei ist das OECD-Land mit dem niedrigsten Wert im Demokratie-Index, Bulgarien hat den niedrigsten Wert in der EU.

Quelle: World Bank, Worldwide Governance Indicators, 2021.

(iii) Demokratien müssen mindestens einen Index so hoch wie der niedrigste Wert eines EU-Staats haben. Bulgarien ist nach dem VA-Index zurzeit der EU-Staat mit dem niedrigsten Index von 0,26. Dahinter steht die Überlegung, dass man EU-Länder nicht vom Handel ausschließen kann und möchte.

(iv) Am weichsten ist die Begrenzung auf diejenigen Länder, die mindestens einen VA-Index wie das OECD-Land mit dem niedrigsten Wert aufweisen. Das ist zurzeit die Türkei (-0,86). Die 38 OECD-Mitglieder verfolgen gemeinsame Ziele, wie einen freien Handel. Neben den expliziten wirtschaftlichen und sozialen Zielen sind faktisch nur Demokratien Mitglieder. Zurzeit fällt die Türkei mit ihrem VA-Wert etwas aus dem Rahmen, das Land mit dem nächsthöheren VA-Wert ist Mexiko (-0,04), dann folgen Kolumbien (0,15), Ungarn (0,39). Bulgarien ist z. B. kein Mitglied.

Die Länder mit dem höchsten BIP der jeweiligen Gruppen werden in Tabelle 1 genannt. Nach dem ersten Kriterium weisen nur weitere 13 Länder einen VA-Wert auf, der mindestens so hoch wie der Wert in Deutschland ist. Das sind Österreich, Schweiz, die Niederlande und Luxemburg, die skandinavischen Staaten, Irland, Kanada und Neuseeland (Spalte (i)). Beim zweiten Kriterium ist Griechenland mit einem VA-Wert von 0,97 das Grenzland, und die Liste umfasst 44 Länder, d. h. es kommen 31 Länder hinzu. Die wichtigsten Länder sind Frankreich, Italien, Großbritannien, die meisten EU-Staaten und Japan. Erst beim dritten Kriterium, also einem VA-Wert von mindestens 0,26 (Bulgarien), sind dann die USA

Tabelle 1

**Ländergruppen nach dem Demokratiegrad 2020**

| (i): VA ≥ 1,38 | (ii): VA ≥ 0,97 | (iii): VA ≥ 0,26 | (iv): VA ≥ -0,86 | (v): VA < -0,86 |
|---|---|---|---|---|
| Kanada, Dänemark, Finnland, Island, Irland, Luxemburg, Niederlande, Neuseeland, Norwegen, Österreich, Schweden, Schweiz | (i) + Australien, Belgien, England, Frankreich, Italien, Japan, Portugal, Spanien, Taiwan, Tschechien | (ii) + Brasilien, Griechenland, Polen, Rumänien, Slowakei, Slowenien, Südafrika, Südkorea, Ungarn, USA | (iii) + Hongkong, Indien, Indonesien, Malaysia, Mexiko, Nigeria, Philippinen, Thailand, Türkei, Singapur | China, Russland, Saudi-Arabien, Ägypten, Iran, VAE, Pakistan, Vietnam, Kasachstan, Irak |

Die Länder sind nach dem VA-Index 2020 sortiert, in Gruppen ii bis v werden nur die jeweils größten Länder aufgeführt.

Quelle: World Bank, World Development Indicators, 2021.

(0,87) dabei.[1] Insgesamt kommen 44 Länder hinzu, demnach werden insgesamt 88 von 202 Ländern erfasst. Beim vierten Kriterium, also einem VA-Wert mindestens so hoch wie in der Türkei (-0,86) sind dann 155 Länder enthalten. Dazu zählen Indien, Mexiko oder die Ukraine. Dann fehlen nur noch die Länder mit einem VA-Wert von weniger als -0,86. Darunter befinden sich noch große Handelspartner wie China mit einem VA-Wert von nur -1,65 (Spalte (v) in Tabelle 1).

## Abwendung von der Welt

Entsprechend unterschiedlich sind die Konsequenzen, je nachdem, welche Länder als ausreichend demokratisch betrachtet werden. Daraus ergibt sich, welche Länder ausgeschlossen werden, die in Tabelle 2 nach den vier eingeführten Definitionen zusammengefasst dargestellt werden. Für jede Gruppe listet Tabelle 2 vier Informationen auf, die einen Eindruck vermitteln, wie groß der Anteil der Welt ist, von dem sich Deutschland durch selbst auferlegte Handelsbeschränkungen abwenden würde: die Zahl der Länder, deren Bevölkerungsgröße, deren Wertschöpfung (gemessen am Bruttoinlandsprodukt) und deren Importe aus Deutschland. Die Bezugsgröße sind alle 202 Länder (ohne Deutschland), für die ein VA-Indexwert verfügbar ist.

Das erste Kriterium für eine hinreichend gute Demokratie ist nicht praktikabel und würde Deutschland isolieren. Das zweite Kriterium ist ebenfalls noch sehr restriktiv, da sich Deutschland von drei Viertel der Länder abwenden würde. Interessanter ist wohl das dritte Kriterium (also das EU-Land mit dem niedrigsten Wert als Mindestanforderung), denn

1 Der VA-Wert der USA ist in den vergangenen Jahren deutlich gesunken. Noch 2016 betrug der VA-Wert der USA 1,11 (D 1,36), 2020 liegt der Wert bei 0,87 (D 1,38). Der Rückgang in den USA hängt mit der Veränderung des politischen Klimas zusammen, das in vielen Fragen, die in die Demokratieindices eingehen, eine Rolle spielt (wie Vertrauen in den Wahlprozess oder gesellschaftlicher Konsens).

Tabelle 2

**Ausgeschlossener Teil der Welt nach Demokratiegrad**

| Dimension | WELT | (i): VA ≥ 1,38 | (ii): VA ≥ 0,97 | (iii): VA ≥ 0,26 | (iv): VA ≥ -0,86 |
|---|---|---|---|---|---|
| Länderzahl | 202 | 190 | 161 | 115 | 48 |
| Bevölkerung | 7,68 Mrd. | 98 % | 92 % | 81 % | 38 % |
| BIP | 77,0 Bn. US$ | 93 % | 73 % | 40 % | 26 % |
| Importe aus Deutschland | 1,2 Bn. US$ | 77 % | 44 % | 20 % | 13 % |

Quelle: World Bank, World Development Indicators, 2021.

hierbei wird die Welt – überspitzt gesagt – in zwei gleich große „gute" und „schlechte" Hälften geteilt. Zum schlechten Teil zählt die Mehrheit von 115 Ländern, die 81 % der Weltbevölkerung ausmachen, aber nur für 40 % der Wertschöpfung auf der Welt und 20 % der deutschen Exporte stehen. Diese Quoten verdeutlichen, dass es sich vor allem um Entwicklungs- und Schwellenländer handelt, zu denen hier auch OECD-Länder wie die Türkei sowie Indien und China zählen. Selbst wenn hierbei „nur" 20 % des bisherigen deutschen Exportmarkts verloren gehen, so geht es doch um rund 40 % der deutschen Exporte außerhalb der EU, um viele schnell wachsende Märkte. Zudem würde Deutschland sich von rund 80 % der Weltbevölkerung abwenden. Diese Selbstbeschränkung des deutschen Außenhandels scheint aus politischen Gründen schwer vorstellbar und auch aus wirtschaftlichen Gründen sehr „teuer", sodass kaum mit solch einer restriktiven Selbstbeschränkung zu rechnen ist.

Demnach bleibt also vor allem das vierte Kriterium als dasjenige, das in der Umsetzung am ehesten praktikabel sein könnte. Natürlich muss dieses – letztlich recht willkürlich gewählte – Kriterium nicht genau so definiert werden, aber es verdeutlicht die Größenordnung. Zur Erinnerung: Der Handel wird auf Länder beschränkt, deren VA-Wert mindestens dem der Türkei entspricht. Diesem Kriterium folgend wird etwa ein Viertel der erfassten Länder vom Handel mit Deutschland ausgeschlossen. Diese Länder stehen immer noch für 38 % der Weltbevölkerung, 26 % des weltweiten BIP und 13 % der deutschen Exporte.

Welche Definition an „ausreichender" Demokratie man auch wählt, der VA-Wert schränkt die Liste der Handelspartner stark ein. Bevor also auf dieser Basis weiter argumentiert wird, soll die Robustheit dieses Maßes geprüft werden, indem sowohl eine einfache Definition von Demokratie als auch ein umfassenderes Maß für gute Governance als Maßstab genutzt wird.

## Robustheit des Maßes für gute Demokratie

Da der VA-Index aufgrund der vielen berücksichtigten Teilindikatoren etwas unübersichtlich scheinen mag, wird

Tabelle 3

**Ausgeschlossener Teil der Welt nach dem Demokratieindex der EIU**

| Dimension | WELT | (i): EIU ≥ 0,85 | (ii): EIU ≥ 0,71 | (iii): EIU ≥ 0,57 | (iv): EIU ≥ 0,35 |
|---|---|---|---|---|---|
| Länderzahl | 179 | 165 | 142 | 114 | 59 |
| Bevölkerung | 7,64 Mrd. | 98 % | 87 % | 64 % | 43 % |
| BIP | 77,0 Bn. US-$ | 91 % | 46 % | 37 % | 28 % |
| Importe aus Deutschland | 1,2 Bn. US-$ | 76 % | 36 % | 19 % | 13 % |

Die Statistiken beziehen sich auf die Verringerung der Teilhabe an Deutschlands Außenhandel.

Quelle: World Bank, World Development Indicators, 2021.

Tabelle 4

**Ausgeschlossener Teil der Welt, gemessen an Governance-Maßen der Weltbank**

| Dimension | WELT | (i): ∅ ≥ 1,40 | (ii): ∅ ≥ 0,41 | (iii): ∅ ≥ 0,14 | (iv): ∅ ≥ -0,47 |
|---|---|---|---|---|---|
| Länderzahl | 195 | 180 | 131 | 117 | 67 |
| Bevölkerung | 7,64 Mrd. | 98 % | 86 % | 84 % | 29 % |
| BIP | 77,0 Bn.US-$ | 90 % | 42 % | 41 % | 7 % |
| Importe aus Deutschland | 1,2 Bn. US-$ | 75 % | 21 % | 19 % | 4 % |

Quelle: World Bank, World Development Indicators, 2021.

nun der Demokratie-Index der EIU (Economist Intelligence Unit) gewählt. Dieser wird nur für 179 Länder ermittelt, insofern sind die absoluten Angaben nicht direkt vergleichbar, aber die relativen Angaben sind es schon. Tabelle 3 listet die Ergebnisse analog zur Tabelle 2 auf. Man sieht, dass die Ergebnisse beider Tabellen sehr ähnlich ausfallen. Konkret würde man beim weichsten Kriterium für Demokratie, also dem niedrigsten Wert eines OECD-Landes (wieder die Türkei), 59 Länder, 43 % der Weltbevölkerung, 28 % des Welteinkommens und 13 % der bisherigen deutschen Exporte vom Handel ausschließen (Spalte (iv) in Tabelle 3).

Zudem wird ein umfassenderes Maß für gut regierte, möglichst demokratische Gesellschaften betrachtet. Die Weltbank erfasst neben dem VA-Index noch fünf weitere Dimensionen für „gutes Regieren“ (Governance). Dies sind (i) politische Stabilität und Abwesenheit von Gewalt, (ii) Regierungseffektivität, (iii) regulatorische Qualität, (iv) Geltung von Gesetzen und (v) Kontrolle von Korruption. Da die Dimensionen dieser Maße alle auf einer Skala von -2,5 bis +2,5 normiert sind, bilden wir für jedes Land den einfachen Durchschnitt der sechs Dimensionen, beziehen somit den VA-Index als eine Dimension mit ein.

Bei diesem Maßstab kommt es also nicht nur auf demokratische Verhältnisse, sondern auch auf funktionierende effiziente Institutionen an. Dieses Maß ist breiter angelegt und „verwässert“ das Maß für Demokratie. Dennoch fällt das Ergebnis in Tabelle 4 wieder ähnlich zur Referenztabelle 2 aus. Es gibt kleine Verschiebungen, vor allem indem undemokratische, aber effizient regierte Länder – wie insbesondere China – bei diesem Maßstab besser abschneiden. Dies wirkt sich dann beim weichsten Kriterium aus, da hier die Gruppe der ausgeschlossenen Länder weit weniger bedeutsam ist (Spalte (iv) in Tabelle 4). In erster Linie liegt dies daran, dass China eine Gruppe „aufrücken“ kann. Dennoch bleibt über alle Spalten und die drei verschiedenen Maße für Demokratie hinweg (VA-Wert, Demokratieindex der EIU und Durchschnitt von sechs Governance-Dimensionen) das Hauptergebnis unberührt: Sofern Deutschland an seine Handelspartner ähnliche Maßstäbe an Demokratie (und gute Governance) wie in Deutschland und Europa anlegt, wird die zulässige Zahl der Handelspartner klein sein, unter Umständen auch sehr klein.

## Entwicklung von Demokratien

Die Lage könnte sich bessern, wenn die Welt möglicherweise immer demokratischer wird. Dann wäre die Zahl der Handelspartner zwar heute noch gering, aber könnte in absehbarer Zukunft erheblich anwachsen. Diese Hoffnung durfte man sicher Anfang der 1990er Jahre mit der Auflösung des früheren „Ostblocks“ hegen. Für die empirische Betrachtung wird das Kriterium 3 genutzt, d. h. alle Länder mit einem VA-Wert von mindestens 0,26 (wie Bulgarien) werden hier als Demokratien klassifiziert. Die Abbildung 2 zeigt für die vergangenen Jahrzehnte aber keinen eindeutigen Trend.

Vielleicht mag der hohe Anteil demokratischer Länder von fast 45 % überraschen, aber die Werte sind von Kleinststaaten getrieben. Der Anteil demokratischer Länder fällt 2020 von 44 % auf 26 %, wenn nur Länder mit über 1 Mio. Einwohner:innen berücksichtigt werden. Ähnlich hängt der Anteil an der Weltbevölkerung, der bei knapp 40 % liegt, entscheidend von der Berücksichtigung Indiens ab. Liegt Indien einmal unter dem Grenzwert, wie 2020, dann halbiert sich der Anteil der Demokratien an der Weltbevölkerung fast auf 20 %. Zusammenfassend scheint es ratsam, eine mögliche politische Entscheidung über zulässige Handelspartner auf Basis des Status quo zu fällen, und nicht auf eine nahe bessere Zukunft zu hoffen.

Eine andere Hoffnung könnte darauf beruhen, dass Demokratien zwar nur einen kleinen Teil der Weltwirtschaft abdecken, aber möglicherweise schneller wachsen. Ein schnelleres Wachstum würde dann ein wenig für die Beschränkung auf diese Ländergruppe kompensieren. In Abbildung 3 ist dieser mögliche Zusammenhang zwi-

Abbildung 2

**Anteile der Welt mit einem Demokratiegrad wenigstens wie Bulgarien**

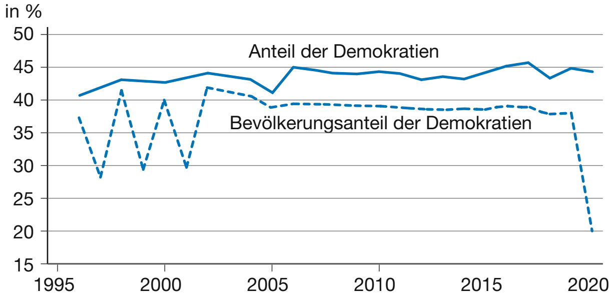

Die Werte beruhen auf dem VA-Index, wobei der Wert von Bulgarien in jedem Jahr als Grenzwert definiert wird. Die durchgezogene Linie zeigt den Anteil der demokratischen Länder an allen Ländern an, die gestrichelte Linie entsprechend den Bevölkerungsanteil der Demokratien.

Quelle: World Bank, World Development Indicators, 2021.

Abbildung 3

**Demokratiegrad und BIP-Wachstum**

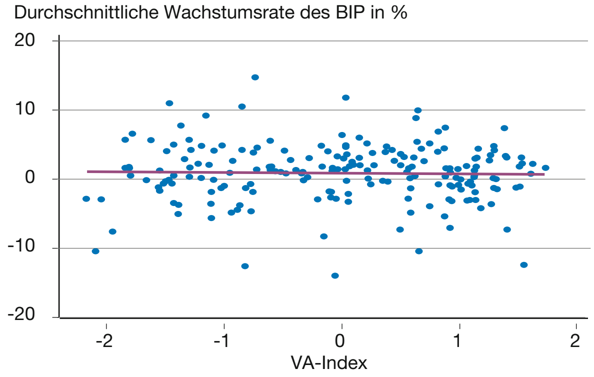

Als Demokratiegrad wird der VA-Index 2020 verwendet. Das BIP-Wachstum ist der Durchschnitt der Jahre 2015-2020. Berücksichtigt werden 202 Länder.

Quelle: World Bank, World Development Indicators, 2021.

schen dem Grad an Demokratie (erfasst über den VA-Wert) und der Wachstumsrate des jeweiligen Landes über die Jahre 2015 bis 2020 abgetragen. Man sieht keinen offensichtlichen Zusammenhang, die Regressionsgerade in Abbildung 3 ist flach. Dies hat verschiedene, nachvollziehbare Ursachen (Entwicklungsstand, Demografie), und limitiert das Potenzial im Außenhandel mit Demokratien.

## Kritische Länder sind nicht marginal

Die Liste der am wenigsten demokratischen Länder gemäß Tabelle 2 umfasst nur Länder, deren demokratische Situation – und passend dazu die Menschenrechtslage – anerkanntermaßen katastrophal ist, nicht nur nach dem VA-Maßstab. Allerdings sind die betreffenden Länder nicht einfach als marginale „Schurkenstaaten“ abzutun, denn die beiden größten Volkswirtschaften in dieser Gruppe sind China und Russland, immerhin beide Vetomächte im UN-Sicherheitsrat. Russland ist gerade als Rohstofflieferant wichtig, China zudem als Absatzmarkt. Wie könnte dann eine Abkopplung von diesen Ländern aussehen?

Eine Art Fallstudie liefern die harten Sanktionen gegenüber Russland als Folge des Kriegs gegen die Ukraine. Dadurch ist der Wirtschaftsaustausch mit Russland stark eingebrochen, mit der großen Ausnahme der russischen Energielieferungen, von denen insbesondere die Gaslieferungen wohl noch über 2022 hinausgehen werden. Sicher war Russland kein demokratischer Handelspartner mit einem VA-Wert von -1,08, der 2022 aufgrund der repressiven Maßnahmen nochmals gesunken ist. Aber die traurige Wahrheit ist auch, dass der deutsche Wirtschaftsminister im März 2022 nach Katar und in die Vereinigten Arabischen Emirate gereist ist, um russisches Gas durch verflüssigtes Gas aus diesen Ländern teilweise zu ersetzen. Deren VA-Werte betragen -1,29 und -1,18, sodass man zwar Handel mit einem kriegerischen Land reduzieren und eventuell vermeiden kann, aber dies gegen Handel mit anderen Problemländern eintauscht. Die beiden arabischen Länder sind wenigstens indirekt in kriegerische Aktivitäten verwickelt und stellen unter dem Gesichtspunkt „gute Demokratie“ keine Verbesserung gegenüber Russland dar.

Was wie ein Einzelfall erscheinen mag, steht für ein generelles Problem. Rohstoffimporte kommen häufig aus wenig demokratischen Ländern. Exemplarisch wird dies am Beispiel von Erdöl deutlich. Die deutsche Hauptbezugsquelle dafür war 2020 Russland mit einem Anteil von über 40 %. Aggregiert man den Anteil der Länder mit VA-Werten unterhalb von Bulgarien bzw. der Türkei, dann lieferten diese Länder 60 % bzw. 53 % der deutschen Rohölimporte (vgl. Abbildung 4).

## China durch Demokratien ersetzen?

Auch die plausible Idee, die starke deutsche Abhängigkeit von China im Außenhandel mit seinem ca. 8 %-Anteil und den gigantischen Direktinvestitionen zu diversifizieren klingt gut. Sie ist aber nicht so einfach umzusetzen, schon gar nicht, wenn man auf Demokratie und Menschenrechte achtet. Hinzu kommt, dass der Handel mit China – anders als der überwiegende Handel mit OECD-Ländern – eine asymmetrische Struktur hat: Er besteht also nicht so sehr im Austausch oft gleichartiger Produkte (wie innerhalb der EU), sondern im Tausch einfacherer gegen höherwertigere Produkte. Insofern kämen als Ersatz für China primär Schwellenländer in Betracht. Schließlich entsprechen der wirtschaftlichen Bedeutung des chinesischen Marktes nicht einzelne Länder, sondern Ländergruppen, um auf ähnliche Größenordnungen zu kommen. Damit kann man vier große Alternativen (und Kombinationen daraus) erwägen: Lateinamerika, Afrika, Indien und Südostasien.

Abbildung 4

**Deutsche Ölimporte nach Demokratiegrad der Lieferländer**

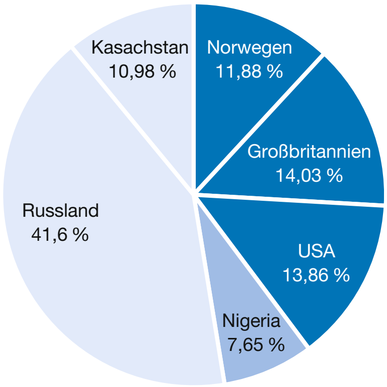

Lieferländer für deutsche Rohölimporte 2020, als Demokratiegrad wird der VA-Index 2020 verwendet.

Quelle: https://de.statista.com/statistik/daten/studie/2473/umfrage/rohoel-import-hauptlieferanten-von-deutschland/#statisticContainer.

Lateinamerika kommt wegen der meist leicht positiven VA-Werte besonders infrage (z.B. Brasilien mit 0,26), weist aber weder eine starke Wirtschaftsdynamik noch besonders offene Märkte auf. Afrika hat zwar in etwa dieselbe Bevölkerungszahl wie China, aber einen viel geringeren Entwicklungsstand, fragmentierte Märkte und meist geringe VA-Werte (z.B. Ägypten -1,49, Nigeria -0,59, Ausnahmefall Südafrika 0,70), sodass es China in der näheren Zukunft nicht annähernd ersetzen kann. Indien hat ebenfalls eine ähnlich große Bevölkerung mit 1,3 Mrd. Menschen, einen VA-Wert von 0,15 und gegenüber Afrika den Vorteil eines weitaus einheitlicheren Markts und einer partiell besseren Entwicklung. Dennoch müsste Indien große Schritte unternehmen, um China in absehbarer Zeit ersetzen zu können (Wagner und Lemke, 2021).

Am realistischsten als Ersatzhandelspartner für China sind demnach umliegende Länder in Südostasien. Diese operieren über den ASEAN-Verbund mit institutionell teils ähnlichen Märkten, zudem ist die Wachstumsdynamik attraktiv. Bei den VA-Werten bieten diese Länder oft nur begrenzte Verbesserungen, wie z.B. Vietnam (-1,38) oder Thailand (-0,81). Vorteilhafter sind die VA-Werte von Malaysia (-0,15), den Philippinen (-0,10) und Indonesien (0,10), um die größten Volkswirtschaften zu nennen – alle Werte liegen allerdings teils deutlich unterhalb des niedrigsten Wertes eines EU-Landes (Bulgarien mit 0,26). Zudem stehen selbst diese fünf größten Volkswirtschaften in Südostasien zusammengenommen 2020 nur für 41 % der chinesischen Bevölkerung und 17 % der chinesischen Wertschöpfung.

## Die Welt der Demokratien ist klein

Wie man es dreht und wendet, die Gesamtmenge aller Demokratien auf der Welt repräsentiert nur eine überschaubare Minderheit der Länder und Bevölkerung, selbst wenn der Anteil am Welteinkommen beträchtlich ist. Ein Blick allein auf diese Märkte und damit die Erwartung, auf Handel mit Nicht-Demokratien leicht verzichten zu können, täuscht aus verschiedenen Gründen: *Erstens* sind die Märkte strukturell verschieden, weil Demokratien meist Länder mit hohem Einkommen sind. *Zweitens* hängt damit zusammen, dass große und schnell wachsende Märkte oft keine Demokratien sind. *Drittens* kommen viele kritische Rohstoffe, zu denen derzeit immer noch Erdöl zählt, aus nicht demokratischen Ländern. Das spricht nicht gegen das Bemühen um Diversifizierung und Begrenzung von Abhängigkeiten, aber es scheint vermessen anzunehmen, man könne den Handel mit nicht demokratischen Ländern problemlos auf demokratische Länder umlenken. Dies hätte hohe ökonomische Kosten zur Folge. Zudem sind weitere Gesichtspunkte zu berücksichtigen, denn letztlich soll das Bemühen um solche Handelslenkung auch die Situation in den wenig demokratischen Ländern verbessern helfen. Es ist allerdings nicht klar, ob ein Ausschluss vom Handel der geeignete Weg ist, um Länder zu mehr Demokratie zu bewegen.
